# Crystal structure of CmABCB1 multi-drug exporter in lipidic mesophase revealed by LCP-SFX

**DOI:** 10.1107/S2052252521011611

**Published:** 2021-12-23

**Authors:** Dongqing Pan, Ryo Oyama, Tomomi Sato, Takanori Nakane, Ryo Mizunuma, Keita Matsuoka, Yasumasa Joti, Kensuke Tono, Eriko Nango, So Iwata, Toru Nakatsu, Hiroaki Kato

**Affiliations:** a Department of Structural Biology, Graduate School of Pharmaceutical Sciences, Kyoto University, 46-29 Yoshida Shimoadachi-cho, Sakyo-ku, Kyoto 606-8501, Japan; b Department of Biological Science, Graduate School of Science, The University of Tokyo, 7-3-1 Hongo, Bunkyo-ku, Tokyo 113-0033, Japan; c Japan Synchrotron Radiation Research Institute, 1-1-1 Kouto, Sayo-cho, Sayo-gun, Hyogo 679-5198, Japan; d RIKEN SPring-8 Center, 1-1-1 Kouto, Sayo-cho, Sayo-gun, Hyogo 679-5148, Japan; e Department of Cell Biology, Graduate School of Medicine, Kyoto University, Yoshidakonoe-cho, Sakyo-ku, Kyoto 606-8501, Japan

**Keywords:** serial crystallography, SFX, protein structures, sample delivery, XFELs, LCP, lipidic mesophase, multi-drug exporters, ABC transporters, *Cyanidioschyzon merolae*, CmABCB1

## Abstract

CmABCB1 is a *Cyanidioschyzon merolae* homolog of human ABCB1, a well known ATP-binding cassette (ABC) transporter responsible for multi-drug resistance in various cancers. Three-dimensional structures of ABCB1 homologs have revealed the snapshots of inward- and outward-facing states of the transporters in action. However, sufficient information to establish the sequential movements of the open–close cycles of the alternating-access model is still lacking. Serial femtosecond crystallography (SFX) using X-ray free-electron lasers has proven its worth in determining novel structures and recording sequential conformational changes of proteins at room temperature, especially for medically important membrane proteins, but it has never been applied to ABC transporters. In this study, 7.7 mono­acyl­glycerol with cholesterol as the host lipid was used and obtained well diffracting microcrystals of the 130 kDa CmABCB1 dimer. Successful SFX experiments were performed by adjusting the viscosity of the crystal suspension of the sponge phase with hy­droxy­propyl methyl­cellulose and using the high-viscosity sample injector for data collection at the SACLA beamline. An outward-facing structure of CmABCB1 at a maximum resolution of 2.22 Å is reported, determined by SFX experiments with crystals formed in the lipidic cubic phase (LCP-SFX), which has never been applied to ABC transporters. In the type I crystal, CmABCB1 dimers interact with adjacent molecules via not only the nucleotide-binding domains but also the transmembrane domains (TMDs); such an interaction was not observed in the previous type II crystal. Although most parts of the structure are similar to those in the previous type II structure, the substrate-exit region of the TMD adopts a different configuration in the type I structure. This difference between the two types of structures reflects the flexibility of the substrate-exit region of CmABCB1, which might be essential for the smooth release of various substrates from the transporter.

## Abstract 2

The 2.22 Å crystal structure of a multi-drug exporter, CmABCB1, was determined by the LCP-SFX method using the XFEL beamline at SACLA. The lipid bilayer-embedded structure revealed new insights about the substrate transport mechanism of CmABCB1.

## 1. Introduction

ATP-binding cassette (ABC) transporters, a large protein family with members in all organisms, mediate translocation of various substrates across membranes by coupling transport to ATP binding and hydrolysis (Rees *et al.*, 2009 ▸; Thomas & Tampé, 2020 ▸; Srikant & Gaudet, 2019 ▸). Among the functionally characterized ABC transporters, multi-drug ABC exporters are responsible for multi-drug resistance (MDR) in various cancers and have been regarded as important protein targets in medical research (Borst & Elferink, 2002 ▸; Robey *et al.*, 2018 ▸). MDR in human cells is caused mainly by three ABC exporters, ABCB1, ABCC1 and ABCG2, which belong to different subfamilies (Robey *et al.*, 2018 ▸). Three-dimensional structures of these transporters, elucidated by extensive X-ray crystallography and cryo-electron microscopy studies, have greatly improved our understanding of their transport mechanisms (Alam *et al.*, 2019 ▸, 2018 ▸; Aller *et al.*, 2009 ▸; Ward *et al.*, 2013 ▸; Kim & Chen, 2018 ▸; Taylor *et al.*, 2017 ▸; Manolaridis *et al.*, 2018 ▸; Johnson & Chen, 2018 ▸, 2017 ▸). These transporters share a common architecture of two transmembrane domains (TMDs) consisting of movable transmembrane (TM) helices dynamically controlled by two nucleotide-binding domains (NBDs), which repeat cycles of binding and hydrolyzing ATP followed by release of ADP and Pi (Thomas & Tampé, 2020 ▸; Srikant & Gaudet, 2019 ▸). Human ABCB1 (also known as P-glycoprotein, MDR1) is typically expressed in barrier tissues and protects cells by exporting xenobiotic compounds (Ambudkar *et al.*, 1999 ▸); however, it also exports major anticancer drugs in the same way. ABCB1 has been proposed to export substrates through an alternating-access mechanism, and this idea has been confirmed by the inward-facing (IF) and outward-facing (OF) structures of human ABCB1 (Alam *et al.*, 2019 ▸; Kim & Chen, 2018 ▸) and its homologs from mice (Alam *et al.*, 2018 ▸; Aller *et al.*, 2009 ▸; Ward *et al.*, 2013 ▸), *Caenorhabditis elegans* (Jin *et al.*, 2012 ▸), and *Cyanidioschyzon merolae* (Kodan *et al.*, 2014 ▸, 2019 ▸). Although the available structures provide snapshots of representative states of the transport cycle, they do not provide enough information to understand the quick structural changes of ABC transporters and their sequential movements. Hence, further development of time-resolved methods is required.

Serial femtosecond crystallography (SFX) using extremely bright X-ray free-electron laser (XFEL) pulses has advanced greatly over the last decade (Chapman *et al.*, 2011 ▸; Weierstall *et al.*, 2014 ▸; Spence, 2017 ▸; Johansson *et al.*, 2017 ▸; Mizohata *et al.*, 2018 ▸). This approach enables unique X-ray diffraction experiments with damage-free data collection, allowing researchers to study the structures of proteins that are susceptible to radiation damage (Kern *et al.*, 2012 ▸; Johansson *et al.*, 2013 ▸; Hirata *et al.*, 2014 ▸; Suga *et al.*, 2015 ▸; Fukuda *et al.*, 2016 ▸). Various experimental phasing methods have proven to be applicable to structural determination (Barends *et al.*, 2014 ▸; Batyuk *et al.*, 2016 ▸; Nakane, Hanashima *et al.*, 2016 ▸; Nakane *et al.*, 2015 ▸; Nass *et al.*, 2016 ▸; Yamashita *et al.*, 2015 ▸, 2017 ▸). The remarkable successes of time-resolved SFX (TR-SFX) made SFX an essential method for unveiling the sequential changes that occur in proteins when they are in action (Tenboer *et al.*, 2014 ▸; Barends *et al.*, 2015 ▸; Pande *et al.*, 2016 ▸; Nango *et al.*, 2016 ▸; Suga *et al.*, 2017 ▸; Tosha *et al.*, 2017 ▸; Shimada *et al.*, 2017 ▸; Stagno *et al.*, 2017 ▸). SFX experiments using lipidic cubic phase (LCP) microcrystals are called LCP-SFX; this method was used to solve the damage-free structure of a G-protein-coupled receptor (GPCR), human serotonin 5-HT_2B_ receptor (Liu *et al.*, 2013 ▸). After the initial success of LCP-SFX, many novel structures of different GPCRs (Caffrey, 2015 ▸; Fenalti *et al.*, 2015 ▸; Zhang *et al.*, 2015 ▸; Zhang, Han *et al.*, 2017 ▸; Zhang, Qiao *et al.*, 2017 ▸; Zhang, Zhao *et al.*, 2017 ▸; Kang *et al.*, 2015 ▸) and a di­acyl­glycerol kinase (Li *et al.*, 2015 ▸) were determined using LCP-SFX, but LCP-SFX is rarely applied to other classes of membrane proteins.

Membrane proteins’ LCP crystals exhibit layered (type I) crystal packing that greatly improves X-ray diffraction, even with micrometre-sized crystals (Caffrey, 2015 ▸). LCP crystals form in a lipid–water mixture, usually with high viscosity; this favors SFX experiments by decreasing the amount of sample required. Ejecting viscous LCP crystals from specially designed high-viscosity sample injectors at a rate of ∼0.1 µl min^−1^ creates a straight stream across the XFEL beam (Weierstall *et al.*, 2014 ▸; Martiel *et al.*, 2019 ▸; Shimazu *et al.*, 2019 ▸). The amount of crystal suspension required is ∼100 times greater when a gas dynamic virtual nozzle injector is used for non-viscous samples (DePonte *et al.*, 2008 ▸; Tono *et al.*, 2015 ▸). To use a high-viscosity sample injector for non-viscous crystals, various viscous delivery media have been tested (Martiel *et al.*, 2019 ▸; Nam, 2019 ▸). Mixing crystals with mineral oil grease (Sugahara *et al.*, 2015 ▸), synthetic grease (Sugahara *et al.*, 2016 ▸), Vaseline (Botha *et al.*, 2015 ▸), agarose (Conrad *et al.*, 2015 ▸), hyaluronic acid (Sugahara *et al.*, 2016 ▸), hy­droxy­ethyl cellulose (HEC) (Sugahara *et al.*, 2017 ▸), nuclear-grade grease (Sugahara *et al.*, 2017 ▸), carb­oxy­methyl cellulose sodium salt (Kovácsová *et al.*, 2017 ▸), pluronic F-127 (Kovácsová *et al.*, 2017 ▸), poly(ethyl­ene oxide) (Martin-Garcia *et al.*, 2017 ▸) or polyacryl­amide (Park *et al.*, 2019 ▸) provides sufficient viscosity and enables data collection by SFX. Among these delivery media, polysaccharide-based hydro­gels have the lowest scattering background (Conrad *et al.*, 2015 ▸; Sugahara *et al.*, 2016 ▸, 2017 ▸; Kovácsová *et al.*, 2017 ▸).

In this study, we performed LCP-SFX experiments using LCP crystals of CmABCB1, the only ABCB1 homolog with high-resolution crystal structures available in both IF and OF conformations (Kodan *et al.*, 2019 ▸, 2014 ▸). We generated well diffracting LCP crystals of CmABCB1 using a mixture of 7.7 mono­acyl­glycerol (MAG) and cholesterol as the host lipid. We successfully collected LCP-SFX data using two different cellulosic thickening reagents, HEC and hy­droxy­propyl methyl­cellulose (HPMC), as the delivery media. The resultant crystal structure of CmABCB1 was in the OF conformation and differed at the substrate exit from the previously reported OF structure (Kodan *et al.*, 2019 ▸), implying that the substrate-exit region is flexible.

## 2. Results

### 2.1. LCP crystals of CmABCB1

To perform SFX experiments at the SPring-8 Ångstrom Free Electron Laser (SACLA) beamline, we used modified crystallization conditions to prepare microcrystals of the type II crystal of the CmABCB1^VVV^ mutant, which carries three point mutations (G277V/A278V/A279V) and easily forms crystals of the IF structure (Kodan *et al.*, 2014 ▸). We obtained microcrystals of CmABCB1^VVV^ with a size of 10–40 µm by increasing the concentration of the precipitant PEG 2000 MME from 12 to 16%. One hundred milligrams of CmABCB1^VVV^ was purified from *Pichia pastoris* cells cultured using a multi-cycle jar-fermentation method, crystallized in batch, and used for data collection at SACLA beamline BL3 (Ishikawa *et al.*, 2012 ▸; Tono *et al.*, 2013 ▸) with a liquid injector system (Tono *et al.*, 2015 ▸). The microcrystals diffracted 7 keV XFEL pulses up to a resolution of 5 Å. However, this was not sufficient for structural determination at atomic resolution, highlighting the limitations of type II membrane protein crystals for SFX experiments.

**Figure 1:**
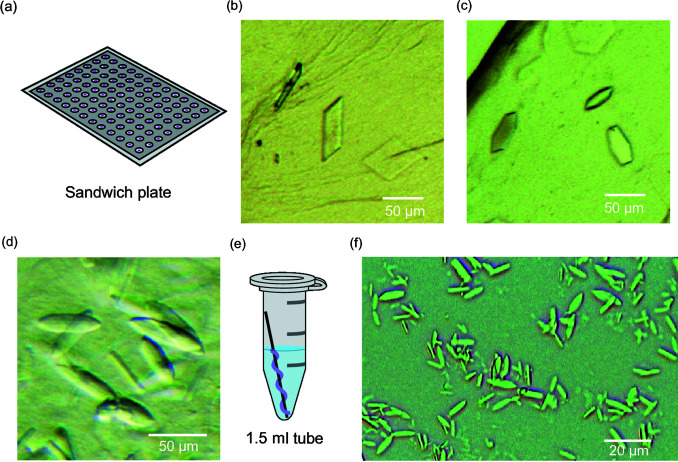
LCP crystals of CmABCB1. (*a*) A diagram of the 96-well glass sandwich plate used for LCP crystallization. (*b*) Rectangular plate crystals, (*c*) hexagonal plate crystals and (*d*) leaf-shaped crystals of CmABCB1^QTA^ formed on the glass sandwich plate. (*e*) A diagram depicting batch LCP crystallization of CmABCB1 in a 1.5 ml tube. (*f*) Microcrystals of CmABCB1^QTA^ formed using the batch method.

We then tried crystallizing CmABCB1 using the LCP method, which has enabled crystallization of many membrane proteins as well diffracting crystals exhibiting type I (layered) crystal packing (Caffrey, 2015 ▸). Even micrometre-sized LCP crystals often provide sufficient diffraction data for structural determination. In these experiments, we replaced CmABCB1 with the CmABCB1^QTA^ mutant carrying two point mutations (Q147A/T381A), which formed type II crystals in both IF and OF conformations (Kodan *et al.*, 2019 ▸). We prepared the LCP mixture using 9.9 MAG and a CmABCB1^QTA^ protein solution containing AMPPNP and Mg^2+^, and screened the crystallization condition with glass sandwich plates [Fig. 1 ▸(*a*)]. Rectangular and hexagonal plate crystals formed under two conditions containing 1,4-butane­diol as the precipitant [Figs. 1 ▸(*b*) and 1 ▸(*c*)]. We then screened salts and found that the hexagonal plate crystals grew to the largest size (50 µm) when 0.2 *M* NH_4_Cl was added to the crystallization solution. The maximum X-ray diffraction of the hexagonal plate crystal reached 4 Å at SPring-8 beamline BL41XU, which encouraged us to conduct further optimization.

We obtained well diffracting LCP crystals when we changed the host lipid of LCP from 9.9 MAG to a mixture of 7.7 MAG and cholesterol. The LCP of 7.7 MAG consists of a thinner lipid bilayer and larger water channels than that of 9.9 MAG (Misquitta *et al.*, 2004 ▸). When we performed screening using the mixture of 7.7 MAG and cholesterol (9:1 *w*/*w*%) as the host lipid, we observed rectangular plate crystals under two conditions and leaf-shaped crystals under one zinc-containing condition [Fig. 1 ▸(*d*)]. After cycles of optimization, high-quality leaf-shaped crystals of CmABCB1^QTA^ were obtained and used for data collection at SPring-8 beamline BL41XU. A complete dataset of 2.7 Å resolution was generated by merging diffraction data from 26 leaf-shaped crystals.

### 2.2. LCP-SFX of CmABCB1

To perform LCP-SFX with CmABCB1^QTA^ LCP crystals, we prepared leaf-shaped crystals using a batch method. LCP paste of CmABCB1^QTA^ and 7.7 MAG/cholesterol was wrapped around a metal wire and soaked in precipitant solution containing 1,4-butane­diol, Tris buffer, and zinc acetate at 20°C [Fig. 1 ▸(*e*)]. Microcrystals of 5–30 µm appeared at high density in three to five days when the concentration of 1,4-butandiol was 26–30% [Fig. 1 ▸(*f*)]. As a result of the high 1,4-butane­diol concentration, the LCP transformed into an oil-like state known as the sponge phase (Caffrey, 2015 ▸). We tried several microcrystal delivery media reported previously (Sugahara *et al.*, 2015 ▸; Nakane, Hanashima *et al.*, 2016 ▸; Suga *et al.*, 2017 ▸; Nango *et al.*, 2016 ▸) to increase the viscosity of our LCP crystal suspension because we wanted to use a high-viscosity cartridge-type (HVC) injector developed in the SACLA beamline (Shimazu *et al.*, 2019 ▸). However, the synthetic grease and the monoolein LCP paste were not able to increase the viscosity of the sponge-phase crystal suspension of CmABCB1^QTA^ to a sufficient degree.

To find the substitute crystal carrier, we tested 23 different thickening reagents generally used as additives of foods, medicines and cosmetics (Table 1 ▸). Thickening reagents were mixed with water in different concentrations (10–40%) and heated at 90°C. Gelatin, polyacrylic acid 1 000 000, HEC and HPMC formed homogeneous transparent gels with sufficient hardness at high concentration. Among these highly soluble reagents, HEC and HPMC were suitable for increasing the viscosity of the sponge-phase crystal suspension of CmABCB1^QTA^ when tested using the HVC injector. Straight streams of the mixtures of CmABCB1^QTA^ crystals and the carriers were stably formed.

Datasets of LCP-SFX of CmABCB1^QTA^ microcrystals were collected at SACLA beamline BL3. HEC and HPMC were dissolved in the crystallization solution of the CmABCB1^QTA^ microcrystals at a concentration of 20–35%. These transparent-gel carrier solutions were mixed with a CmABCB1^QTA^ sponge-phase microcrystal suspension at a 1:1 ratio with the syringes and coupler usually used for LCP preparation. The resultant crystal mixture, containing 10–12.5% HEC or 12.5–17.5% HPMC, was loaded onto the HVC injector and ejected at 0.4 µl min^−1^ to form a straight stream. Four diffraction datasets were collected using 30 Hz XFEL pulses for 70–140 min for each condition (Table 2 ▸). We collected 122 070, 157 812, 183 424 and 258 430 images for the 10% HEC, 12.5% HEC, 12.5% HPMC and 17.5% HPMC datasets, respectively. Comparison of the statistics of the 12.5% HEC and 12.5% HPMC datasets implied that HPMC was more suitable for data collection of CmABCB1 LCP microcrystals because both the hit rate and the number of indexed images were better. The statistics improved when we decreased the concentration of HEC to 10% or increased the concentration of HPMC to 17.5% (Table 2 ▸). The dataset with the highest resolution (2.22 Å) was obtained using 17.5% HPMC as the carrier.

### 2.3. LCP crystal structure of CmABCB1

We analyzed the datasets of the CmABCB1^QTA^ crystals and determined the crystal structure of leaf-shaped LCP crystals of CmABCB1^QTA^ using the molecular replacement method. No significant difference was observed among the structures determined using the available datasets. Therefore, we performed the final refinement using the 17.5% HPMC dataset, which had the highest resolution (Table 2 ▸).

**Figure 2:**
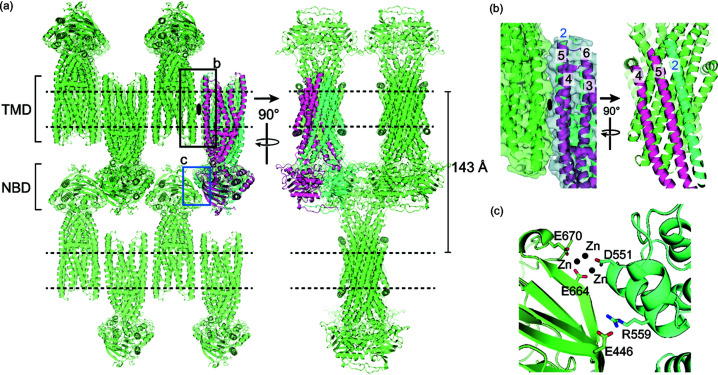
Type I crystal packing of the CmABCB1^QTA^ OF crystal structure. (*a*) Crystal packing of CmABCBC1^QTA^ molecules shows alternating layers of antiparallel homodimers within the crystal. (*b*) An interface formed by the TM2, TM4 and TM5 helices of two CmABCB1^QTA^ dimers. (*c*) Major interactions at the NBD interface.

In the crystal, CmABCB1^QTA^ forms homodimers in an OF conformation [Fig. 2 ▸(*a*)]. The asymmetric unit contains only one CmABCB1^QTA^ molecule. The two CmABCB1^QTA^ molecules forming a dimer are related to each other with twofold rotational symmetry. In this OF CmABCB1 structure, two NBDs are attached to each other, sandwiching two AMPPNP:Mg^2+^ complexes between them. Twelve TM helices from both molecules formed a cup-shaped structure with an opening towards the opposite side of the NBD.

As expected, CmABCB1^QTA^ dimers packed in layers and formed type I crystals. The TMDs of the dimers were aligned in the same plane when adjacent molecules were displayed by applying the symmetry operation [Fig. 2 ▸(*a*)], suggesting that the TMDs of CmABCB1 stayed in lipid bilayers of 7.7 MAG/cholesterol in the crystal. Dimers next to each other were packed in an antiparallel orientation. Transmembrane helix 2 (TM2), TM4 and TM5 of one CmABCB1 molecule interacted with the same three helices of the adjacent molecule in an antiparallel orientation [Fig. 2 ▸(*b*)]. This interface of TMs contained many hydro­phobic residues packed against each other in the lipid bilayer, whereas these hydro­phobic residues were covered by detergent micelles in the type II crystal reported previously (Kodan *et al.*, 2019 ▸). The hydro­philic regions of CmABCB1 dimers also interacted with each other via NBDs in the large aqueous space between the lipid bilayers. The distance between the lipid bilayers was 143 Å. A salt bridge between Arg559 and Glu446 and a zinc cluster containing three zinc ions interacting with three acidic residues (Asp551, Glu664 and Glu670) from two adjacent NBDs mediated the interactions between CmABCB1 dimers [Fig. 2 ▸(*c*)]. These head-to-head and tail-to-tail interactions determined the crystal packing and generated the well diffracting LCP crystals.

### 2.4. Flexible nature of the substrate-exit region

**Figure 3:**
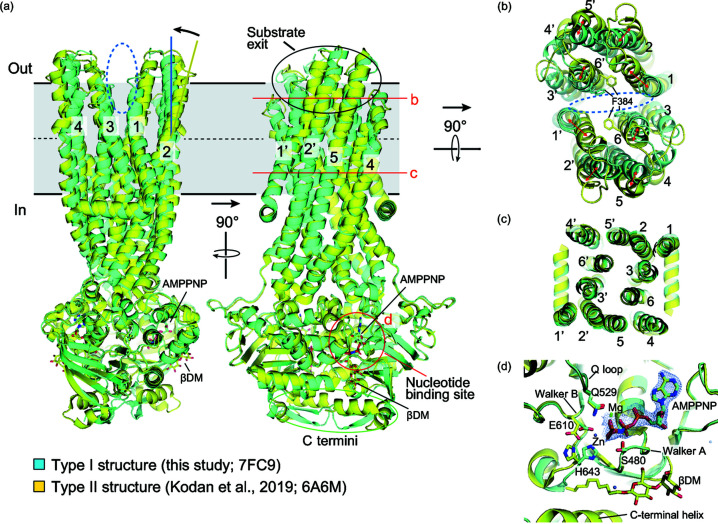
Structural comparison of the type I and type II OF crystal structures of CmABCB1^QTA^. (*a*) Structure alignment of the two types of crystal structures. Black solid lines indicate the boundaries of the lipid bilayer. Black dashed lines indicate the middle of the lipid bilayer. Red solid lines and a red ellipse indicate regions enlarged in (*b*), (*c*), and (*d*). Ellipses with blue dashed lines in (*a*) and (*b*) indicate the cleft of the substrate exit. (*b*) Displacement of the TM helices at the outer leaflet region constituting the substrate exit. (*c*) The inner leaflet part of the TM helices exhibits little difference. (*d*) Nucleotide-binding sites of the two structures. The βDM molecule belongs to the type II structure and the Zn ion belongs to the type I structure. The blue mesh shows a 2*F*_o_ − *F*_c_ electron-density map of AMPPNP in the type I structure, contoured at the 1.5σ level.

Structural alignment of the OF structure from this study with an OF structure from a previous report (Kodan *et al.*, 2019 ▸) revealed the flexibility of the substrate-exit region of CmABCB1. In the two OF structures, two NBDs of CmABCB1 bound tightly to each other, assisted by the binding of the unhydrolyzable ATP analog, AMPPNP. This association of NBDs generated an overall conformational change through the TMD helices and opened the substrate exit at the extracellular site [Fig. 3 ▸(*a*)]. We observed little difference from the NBD region to the lower half of the TM region when we superimposed the two OF structures [Figs. 3 ▸(*a*)–3 ▸(*c*)]. However, the upper half of the TM region exhibited significant differences [Figs. 3 ▸(*a*)–3 ▸(*c*)]. Parts of helices TM1, TM2, TM5 and TM6 constituting the substrate exit exhibited changes in orientation and 2–6 Å displacements [Figs. 3 ▸(*a*) and 3 ▸(*b*)]. The cleft of the substrate exit was slightly narrower in the type I structure but still showed a clear opening, as indicated by the sidechains of Phe384, an important residue that blocks substrate molecules when CmABCB1 is in an IF conformation (Kodan *et al.*, 2019 ▸). These movements were caused by type I crystal packing in the lipid bilayer, in the sense that helices TM2, TM4 and TM5 from two adjacent molecules pushed against each other and became more vertical relative to the lipid bilayer [Figs. 2 ▸(*b*) and 3 ▸(*a*)]. TM1 and TM6 are connected to TM2 and TM5, respectively, by the extracellular loops, and their positions were consequently affected. In the previous type II structure, the substrate exit was surrounded by the detergent micelle and adopted a more opened conformation.

The C-terminal helix in the previous structure became a β-strand in the new structure and formed a small β-sheet with the same β-strand of the other subunit [Fig. 3 ▸(*a*)]. One βDM molecule bound closely to the C-terminal helix in the previous structure, which might have stabilized the helical conformation [Figs. 3 ▸(*a*) and 3 ▸(*d*)].

In the new structure, AMPPNP and Mg^2+^ bound at the same position of the nucleotide-binding pocket formed by the two NBDs [Figs. 3 ▸(*a*) and 3 ▸(*d*)]. The Walker A, Walker B and Q-loop motifs also showed little difference between the two structures [Fig. 3 ▸(*d*)]. A zinc ion was found next to the γ-phosphate of the AMPPNP in the new structure, where it interacted with Glu610 and His643 [Fig. 3 ▸(*d*)].

## 3. Discussion

In this study, we performed an LCP-SFX experiment on the CmABCB1 multi-drug transporter and solved the type I crystal structure in the OF conformation. This new structure confirmed the overall fold of the OF conformation reported previously (Kodan *et al.*, 2019 ▸) and provided new insights about the flexibility of the substrate exit. CmABCB1 dimer transports diverse small-molecule substrates from the cytosol across the plasma membrane by coupling the energy of ATP hydrolysis to the repetition of IF and OF conformational changes (Kodan *et al.*, 2019 ▸). This kind of alternating-access mechanism is shared by other ABCB1 homologs and ABC exporters (Thomas & Tampé, 2020 ▸; Srikant & Gaudet, 2019 ▸). Substrate molecules enter CmABCB1 from the cytosolic side of the IF conformation, are pushed upward to the substrate exit as NBDs associate and diffuse outward from the substrate exit when water molecules flow in. We hypothesize that the structural rigidity of CmABCB1 from the NBD to the lower part of the TM region and the flexibility of the substrate exit are both essential for the multi-drug export activity: the rigidity is required to transfer the conformational changes, whereas the flexibility is required to release the substrate. The flexible nature of the substrate-exit region was also observed in the OF structure of human ABCB1 (Kim & Chen, 2018 ▸). In this structure, the extracellular regions that form the substrate exit exhibit less defined electron-microscopy density and higher *B* factors relative to the rest of the structure (Kim & Chen, 2018 ▸), which is consistent with our observations for CmABCB1.

The CmABCB1 LCP crystal is suitable for future TR-SFX studies. The OF conformation of CmABCB1 is in a high-energy state (Srikant & Gaudet, 2019 ▸). The transporter will quickly return to the IF conformation as soon as ATP is hydrolyzed. If the hydrolysis can be induced in a controlled manner, we might be able to track the movement by coupling hydrolysis to the overall conformational change of the transporter. Good methods to induce ATP hydrolysis are essential to achieving this goal, and development of these techniques requires additional investigation.

LCP-SFX has been used to determine the structures of many GPCRs (Liu *et al.*, 2013 ▸; Kang *et al.*, 2015 ▸; Ishchenko *et al.*, 2017 ▸; Johansson *et al.*, 2019 ▸; Zhang, Han *et al.*, 2017 ▸) and for analyzes of time-resolved structural changes (Nogly *et al.*, 2018 ▸, 2016 ▸; Nango *et al.*, 2016 ▸), but has never been applied to the study of ABC transporters, which usually possess large extramembrane regions including NBDs. This structural feature could be a disadvantage for LCP crystal formation. However, several studies have reported that protein complexes with large extramembrane regions could form LCP crystals (Rasmussen *et al.*, 2011 ▸; Ishchenko *et al.*, 2017 ▸; Asada *et al.*, 2018 ▸). We successfully obtained LCP crystals of CmABCB1 using 9.9 MAG (monoolein) as the host lipid but the best crystals were obtained when we changed the host lipid to a 7.7 MAG/cholesterol mixture. Crystals formed mainly under conditions containing 1,4-butane­diol, which induces the sponge phase (Caffrey, 2015 ▸). Our leaf-shaped crystals also formed in the oil-like sponge-phase solution. This supports the assumption that the sponge phase is preferable for type I crystal formation when using membrane proteins with large extramembrane regions (Caffrey, 2015 ▸).

Stable stream formation of CmABCB1 sponge-phase crystals at the SACLA beamline was achieved using the thickening reagents HEC and HPMC. HEC was previously used as the crystal carrier for SFX experiments on soluble protein crystals (Sugahara *et al.*, 2017 ▸). One advantage of a cellulose-based delivery medium is the low background scattering (Nam, 2019 ▸). HEC and HPMC are both highly soluble and therefore very useful for improving viscosity when the crystal suspension cannot be easily concentrated by centrifugation. Mixing 20–25% HEC or 30–35% HPMC with equal volumes of sponge-phase crystal suspension produced an adequate paste for stable stream formation using the HVC injector (Shimazu *et al.*, 2019 ▸) at the SACLA beamline. We obtained high-quality datasets using both HEC and HPMC; the HPMC datasets were slightly better than the HEC datasets in terms of maximum resolution. Although we need to perform further systematic evaluation of HPMC as a crystal carrier, we can conclude that HPMC is comparable with HEC for delivering microcrystals in SFX experiments and that HPMC was superior to HEC in the case of CmABCB1 LCP microcrystals.

## 4. Materials and methods

### 4.1. Protein expression and purification

CmABCB1^QTA^ was expressed in *P. pastoris* strain SMD1163. Plasmid pPICZA-CmABCB1(QTA)-FLAG-6His was generated by sub-cloning the coding sequence of CmABCB1 with a C-terminal FLAG-6His tag from pABC3-CmABCB1-FLAG-His (Kodan *et al.*, 2014 ▸) into pPICZA using the EcoRI and SalI sites (Thermo Fisher Scientific). The QTA mutation (Q147A/T381A) was introduced using primers reported previously (Kodan *et al.*, 2019 ▸) by PCR-based site-directed mutagenesis. The resultant open reading frame expresses the 696 amino acid CmABCB1 with an additional amino acid sequence, SGRDYKDDDDKHHHHHH, at the C terminus.

*P. pastoris* SMD1163 cells containing the integrated sequence pPICZA-CmABCB1(QTA)-FLAG-6His were cultured in a jar fermenter with a BIOFLO310 system (New Brunswick Scientific), and protein expression was induced by addition of methanol. We modified the fed-batch fermenter protocol of Kodan *et al.* (2014 ▸) to enable sequential culture by seeding the new culture with one tenth of the harvested cells. *P. pastoris* cells were cultured in 1 l YPD medium in a baffled flask and grown at 30°C until the OD reached 5 (600 nm). The cells were separated from the medium by centrifugation and resuspended in 4 l fermentation medium [0.4*M* KH_2_PO_4_, 2%(*w*/*v*) Bacto yeast extract, 4%(*w*/*v*) Bacto peptone, 2.68%(*w*/*v*) yeast nitro­gen base without amino acids, 0.5%(*w*/*v*) sorbitol, 2%(*w*/*v*) hicasamino acids (Daigo), 0.3%(*v*/*v*) methanol, 4 × 10^−5^%(*w*/*v*) biotin, 100 µg ml^−1^ zeocin, 100 µg ml^−1^ ampicillin sodium]. Temperature was maintained at 25°C and the concentration of dissolved oxygen was maintained at 40% air saturation. To optimize both cell growth and the expression of CmABCB1, methanol concentration was maintained at 0.3% by automatic control. After 48 h of culture, cells were harvested from 3.6 l of medium, and the remaining 0.4 l culture was combined with 3.6 l of newly prepared fermentation medium for the second cycle of fermentation. This process could be repeated several times. Each harvest yielded ∼300 g of cells.

CmABCB1^QTA^ was purified as previously described (Kodan *et al.*, 2014 ▸) with minor modifications. The cell pellet (300 g) was suspended in lysis buffer [20 m*M* Tris–HCl pH 7.0, 150 m*M* NaCl, protease inhibitor cocktail (Roche)] to a volume of 750 ml and disrupted with an EmulsiFlex homogenizer (Avestin). The cell debris was removed by centrifugation at 1500*g* for 15 min and the membrane fraction was collected by centrifugation at 100 000*g* for 60 min. Protein solubilization was performed by homogenizing the membrane with buffer containing 20 m*M* Tris–HCl pH 7.0, 300 m*M* NaCl, 20 m*M* imidazole, 1%(*w*/*v*) NIKKOL BL-9EX (Wako) and protease inhibitor cocktail (Roche), followed by incubation for 1 h at 4°C. Insoluble material was removed by centrifugation at 100 000*g* for 60 min. CmABCB1^QTA^ with the FLAG-6His tag was purified by Ni-affinity chromatography with Ni-IMAC resin (Bio-Rad) and eluted with elution buffer containing 20 m*M* Tris–HCl pH 7.0, 300 m*M* NaCl, 500 m*M* imidazole, and 0.2%(*w*/*v*) *n*-decyl-β-d-malto­pyran­oside (βDM; Anatrace). The protein solution was subjected to trypsin digestion with 5 µg trypsin per 1 mg CmABCB1 (27°C, 30 min) to remove the N-terminal 92 residues and the FLAG-6His tag of CmABCB1 as described previously (Kodan *et al.*, 2014 ▸). The final step of size-exclusion chromatography (SEC) was performed using a HiLoad 16/600 Superdex 200 pg column (GE Healthcare) with SEC buffer containing 20 m*M* Tris–HCl pH 7.0, 150 m*M* NaCl and 0.2%(*w*/*v*) βDM. Peak fractions containing CmABCB1^QTA^ were concentrated to 30 mg ml^−1^. Approximately 6 mg of CmABCB1^QTA^ was obtained from 1 l culture of fed-batch fermentation.

CmABCB1^VVV^ was expressed in *P. pastoris* strain SMD1163 and purified in the same manner as CmABCB1^QTA^. Plasmid pPICZA-CmABCB1(VVV)-6His was generated by sub-cloning the coding sequence of CmABCB1 with the C-terminal 6His tag from pABC3-CmABCB1-FLAG-His (Kodan *et al.*, 2014 ▸) into pPICZA. The VVV mutation (G277V/A278V/A279V) was introduced by PCR-based site-directed mutagenesis as previously reported (Kodan *et al.*, 2014 ▸). The resultant open reading frame expresses the 696 amino acid CmABCB1 with an additional amino acid sequence, VDHHHHHH, at the C terminus.

### 4.2. Type II microcrystal production

Protein solution containing 10 mg ml^−1^ CmABCB1^VVV^ in SEC buffer was combined with crystallization solution containing 16% PEG 2000 MME, 0.1 *M* magnesium nitrate and 0.1% βDM at a volume ratio of 1:1. After gentle mixture and incubation at 10°C for 5 h, microcrystals were grown to 10–40 µm.

### 4.3. LCP crystallization and LCP microcrystal production

LCP crystallization trials of CmABCB1^QTA^ were performed as previously described (Caffrey & Cherezov, 2009 ▸). Twenty microlitres of protein solution of CmABCB1^QTA^ at 30 mg ml^−1^ was incubated for 1 h with 10 m*M* adenosine 5′-(β,γ-imido)triphosphate lithium salt hydrate (AMPPNP, Sigma) and 20 m*M* MgCl_2_ at 20°C. The protein samples were mixed with 30 µl 1-oleoyl-rac-glycerol (monoolein or 9.9 MAG, Sigma) using 100 µl gastight syringes (Hamilton). Fifty nanolitres of the mixture was dispensed onto each well of a 96-well glass sandwich plate (Hampton Research) and covered with 800 nl of crystallization solution. For the initial screening, crystallization conditions were explored at 20°C using the Wizard Cubic LCP kit (Rigaku). Rectangular plate crystals were observed under condition #26 (20% 1,4-butane­diol, 0.1 *M* Tris–HCl pH 8.5, 0.2 *M* MgCl_2_) and hexagonal plate crystals were observed under condition #27 (20% 1,4-butane­diol, 0.1 *M* citrate pH 5.5, 0.2 *M* NaCl). StockOptions Salt (Hampton Research) was used to explore the effects of 48 different salts. Addition of ammonium chloride yielded the best rectangular plate crystals with crystallization solution containing 25% 1,4-butane­diol, 0.1 *M* citrate pH 5.0 and 0.2 *M* NH_4_Cl. Crystals from this condition diffracted up to 4 Å at SPring-8 synchrotron beamline BL41XU.

Leaf-shaped LCP crystals formed when a mixture of 1-(7Z-tetradecenoyl)-rac glycerol (7.7 MAG, Avanti) and cholesterol (9:1 ratio) was used as the host lipid. To achieve complete solubilization of cholesterol in 7.7 MAG, the mixture of 7.7 MAG and cholesterol was sonicated in a Bioruptor UCD-200 (Diagenode) until the mixture turned into a transparent solution. When we repeated the screen, solution #25 from the Wizard Cubic LCP kit [20% 1,4-butane­diol, 0.1 *M* Tris–HCl pH 7.0, 0.2 *M* Zn(OAc)_2_] produced leaf-shaped crystals. Two other conditions (#23 and #81) yielded rectangular plate crystals. After optimization, the largest leaf-shaped crystal (90 µm) formed in a crystallization solution containing 15% 1,4-butane­diol, 0.1 *M* Tris–HCl pH 7.3 and 0.2 *M* Zn(OAc)_2_.

Microcrystals of leaf-shaped CmABCB1^QTA^ crystal were generated by incubation of 50 µl LCP containing CmABCB1^QTA^ and 7.7 MAG/cholesterol as described above in a 1.5 ml tube with 700 µl of crystallization solution containing 26–30% 1,4-butane­diol, 0.1 *M* Tris pH 7.9 and 0.2 *M* Zn(OAc)_2_. To facilitate LCP transfer and positioning in the tube, the LCP paste was wrapped around a steel wire when placed in the crystallization solution inside the tube. Microcrystals of 5–30 µm in length appeared after three to five days of incubation at 20°C. The LCP crystals on the wire were transferred to a new tube and separated from the crystallization solution by holding the wire with tweezers. Some LCP crystals detached from the wire and were left floating on top of the crystallization solution. These crystals were collected using a micropipette.

### 4.4. Data collection at synchrotron

CmABCB1^QTA^ LCP crystals were fished from the sandwich plates using MicroMeshes (MiTeGen) and were cryo-cooled with liquid nitro­gen. All datasets of X-ray diffraction images were collected at SPring-8 beamline BL41XU (Hasegawa *et al.*, 2013 ▸) using the helical data-collection method provided by the beamline’s system. The amount of radiation damage was estimated using *RADDOSE3D* (Zeldin *et al.*, 2013 ▸). A 460 µm Al attenuator was used to collect diffraction images with average radiation damage of <10 MGy. Diffraction patterns of a 15–50° wedge were collected from each crystal at 100 K at a wavelength of 1.0000 Å. The exposure time was 1 s per frame and the oscillation range was 1° per frame. Datasets of leaf-shaped LCP crystals were collected from 105 crystals. The best 26 datasets were chosen and merged for further analysis.

### 4.5. LCP-SFX data collection at SACLA

The LCP mixture of CmABCB1^QTA^, 7.7 MAG and cholesterol adopted an oil-like low-viscosity state after incubation with crystallization solution containing 1,4-butane­diol. Microcrystals formed in the low-viscosity LCP mixture within three to five days. To increase the viscosity of the crystal suspension, we sought to identify suitable crystal carriers from among 23 different commercially available thickening agents (see Table S1 of the supporting information). Powders of these thickening agents were combined with water at various concentrations, heated at 90°C for 1 h and incubated at 25°C for one day. Air bubbles were removed by centrifugation. Four highly soluble thickening agents (HEC, HPMC, polyacrylic acid 1 000 000, gelatin) were further evaluated for the ability to form a continuous stream using the HVC injector (Shimazu *et al.*, 2019 ▸) at the SACLA beamline.

HEC and HPMC were able to support continuous streams and were therefore chosen for LCP-SFX experiments. HEC or HPMC was dissolved in the crystallization solution [26% 1,4-butane­diol, 0.1 *M* Tris pH 7.9, 0.2 *M* Zn(OAc)_2_] at concentrations ranging from 20 to 35%. These paste-like solutions were combined at 1:1 ratio with CmABCB1^QTA^ LCP crystals using two syringes and a coupler as described previously (Conrad *et al.*, 2015 ▸). The viscosity-adjusted crystal suspension was placed in the HVC injector, and SFX experiments were performed at SACLA beamline BL3 (Ishikawa *et al.*, 2012 ▸; Tono *et al.*, 2013 ▸). A continuous stream of crystal suspension was created at a flow rate of 0.4 µl min^−1^. Diffraction images were collected at 25°C with XFEL pulses of 30 Hz at 7.0 keV. The beam size was adjusted to 1.5 × 1.5 µm. Diffraction patterns were recorded using a multiport CCD detector with eight sensor modules (Kameshima *et al.*, 2014 ▸) at a sample-to-detector distance of 50 mm. The statistics of data collection are summarized in Table 2 ▸. Raw XFEL diffraction images for this article have been deposited in the Coherent X-ray Imaging Data Bank (CXIDB) (ID 193).

### 4.6. Data reduction and structure determination

LCP-SFX data collection was monitored using the on-the-fly data-processing pipeline for SFX at SACLA (Nakane, Joti *et al.*, 2016 ▸). Crystal hits were identified by *Cheetah* (Barty *et al.*, 2014 ▸) and processed with *CrystFEL* 0.9.0 (White *et al.*, 2012 ▸). Diffraction spots were located by the *peakfinder8* algorithm and indexed with *XGANDALF* (Gevorkov *et al.*, 2019 ▸). Integrated intensities were merged by *partialator* in the *CrystFEL* suite with scaling but without partiality corrections. Synchrotron diffraction data were processed using *XDS* (Kabsch, 2010 ▸).

The initial phases were determined by molecular replacement using *Phaser* (McCoy *et al.*, 2007 ▸). The crystal structure of the CmABCB1 outward-open form (PDB entry 6a6m; Kodan *et al.*, 2019 ▸) was used as the search model. Structure building and refinement were performed with *Coot* (Casañal *et al.*, 2020 ▸) and *REFMAC*5 (Kovalevskiy *et al.*, 2018 ▸). Data collection and processing information are summarized in Table 2 ▸. *PyMOL* was used to make figures.

## Supplementary Material

Table S1. DOI: 10.1107/S2052252521011611/cw5035sup1.xlsx

PDB reference: CmABCB1 in lipidic mesophase by LCP-SFX, 7fc9

Raw XFEL diffraction images. CXIDB ID 193.: https://doi.org/10.11577/1835522

## Figures and Tables

**Table 1:** Water solubility of 23 thickening agents S, soluble; IS, insoluble.

	Thickening agents	Concentration (*w*/*v*%)			
		10	20	30	40
1	Aluminium stearate, mono	IS	IS	IS	IS
2	Amylopectin hydrate	IS	IS	IS	IS
3	Carb­oxy­methyl cellulose, sodium salt (*n* = ∼1050)	S	IS	IS	IS
4	Carb­oxy­methyl cellulose, sodium salt (*n* = ∼500)	S	IS	IS	IS
5	Gelatin, from bovine bone	S	S	S	S
6	Guar gum	IS	IS	IS	IS
7	HEC	S	S	S	S
8	Hy­droxy­propyl cellulose 1000–5000 cP (1 cP = 10^−3^ Pa s = 1 mPa s)	S	IS	IS	IS
9	Hy­droxy­propyl cellulose 150–400 cP	S	IS	IS	IS
10	Hy­droxy­propyl cellulose 6.0–10.0	S	IS	IS	IS
11	HPMC	S	S	S	S
12	Locust bean gum	S	S	S	S
13	Methyl cellulose 400	S	IS	IS	IS
14	Methyl cellulose 4000	S	IS	IS	IS
15	Methyl cellulose 50	S	S	S	IS
16	Poly(vinyl alcohol) 1000, completely hydrolyzed	S	S	S	S
17	Polyacrylic acid 1000000	S	S	S	S
18	Polyacrylic acid 25000	S	S	S	S
19	Sodium alginate 500–600	S	IS	IS	IS
20	Sodium alginate 80–120	S	IS	IS	IS
21	Starch, soluble	S	S	S	IS
22	Xanthan gum	IS	IS	IS	IS
23	κ-Carrageenan	IS	IS	IS	IS

**Table 2:** Data-collection and refinement statistics Values in parentheses are for the highest-resolution shell.

	LCP crystals from sandwich plates	LCP microcrystals (10% HEC)	LCP microcrystals (12.5% HEC)	LCP microcrystals (12.5% HPMC)	LCP microcrystals (17.5% HPMC)
Data collection					
Beamline	SPring-8 BL41XU	SACLA BL3	SACLA BL3	SACLA BL3	SACLA BL3
Crystal size (µm)	∼90	5–30	5–30	5–30	5–30
Wavelength (Å)	1.0000	1.771	1.771	1.771	1.771
Beam size (µm)	12 × 4	1.5 × 1.5	1.5 × 1.5	1.5 × 1.5	1.5 × 1.5
Beam-time used (min)	–	70	90	110	140
No. of collected images	715 (26 crystals)	122070	157812	183424	258430
No. of hits/indexed patterns	–	37873/27725	41680/21580	121970/92365	143948/118668
No. of total reflections	535610	17220391	13020234	75672715	102801197
Space group	*C*222_1_	*C*222_1_	*C*222_1_	*C*222_1_	*C*222_1_
Unit-cell parameters *a, b, c* (Å)	73.7, 285.2, 85.6	74.5, 284.7, 86.4	74.2, 283.5, 86.0	74.8, 285.3, 86.7	74.9, 285.9, 86.8
Resolution range (Å)	48.16–2.70 (2.74–2.70)	45.24–2.47 (2.51–2.47)	45.05–2.57 (2.67–2.62)	45.37–2.37 (2.41–2.37)	45.45–2.22 (2.26–2.22)
No. of unique reflections	24751 (1082)	34355 (1693)	28476 (1408)	39210 (1933)	47845 (2335)
Completeness (%)	97.7 (99.1)	100 (100)	100 (100)	100 (100)	100 (100)
Multiplicity	21.6 (21.8)	501 (239)	457 (184)	1930 (848)	2149 (591)
〈*I*/σ(*I*)〉	13.08 (1.92)	7.21 (1.59)	6.89 (1.40)	11.00 (1.65)	12.37 (1.43)
*R*_meas_ (%)	21.2 (161.7)	–	–	–	–
*R*_split_ (%)	–	10.13 (71.16)	11.18 (78.85)	6.57 (72.46)	5.67 (84.84)
CC_1/2_ (%)	99.6 (39.6)	–	–	–	–
CC* (%)	–	99.77 (84.85)	99.75 (82.78)	99.94 (84.32)	99.96 (77.09)
					
Refinement					
No. of reflections					47761
No. in test set					2491
*R*_work_/*R*_free_ (%)					18.5/21.0
No. of atoms, protein/ligand/water					4448/48/152
*B* factor (Å^2^), protein/ligand/water					63.8/42.3/54.9
R.m.s.d., bonds (Å)/angles (°)					0.0041/1.245
Ramachandran plot statistics (%)					
Favored/allowed/disallowed					97.4/2.6/0.0
